# The Psychological Consequences of Advice Giving: Advisors’ Moral Self-Evaluations, Memory Errors and the Impact of Prosocial Cost

**DOI:** 10.3390/bs16050730

**Published:** 2026-05-09

**Authors:** Xiuxin Wang, Mengyao Tian, Xiaohan Hu, Yifan Shen

**Affiliations:** School of Psychology, Qufu Normal University, Qufu 273165, China

**Keywords:** moral self, conflict of interest, advice-giving, memory error

## Abstract

When advisors’ interests conflict with those of recipients, they may give selfish advice. Previous research has extensively examined the factors influencing advisors’ tendencies to offer self-serving advice but has rarely investigated the psychological consequences of such advice-giving. Based on moral self-threat theory, this study investigated advisors’ moral self-evaluations and memory errors, focusing on the role of prosocial cost. Across three studies, participants played the role of an advisor, making repeated choices between selfish and altruistic options in scenarios with either high or low prosocial costs. They later recalled their choices. The results consistently showed that the number of prosocial advice choices was positively associated with advisors’ moral self-evaluations, regardless of whether the prosocial cost was high or low, a pattern consist with the moral self-threat. Furthermore, participants who made few prosocial advice choices (i.e., relatively selfish advisors) showed self-serving memory errors, recalling themselves as more altruistic than they were. Interestingly, participants who made many prosocial advice choices (i.e., relatively altruistic advisors) showed no memory bias under low prosocial cost but displayed self-defeating errors under high prosocial cost, inaccurately recalling that they gave less altruistic advice than they actually did. These findings extend our understanding of the psychological consequences of advice-giving and highlight the novel phenomenon of self-defeating memory errors.

## 1. Introduction

Please imagine a banker who has two possible loans to recommend to a client. Loan A is optimal for the client; however, the banker will obtain little benefit from it. Loan B is not optimal for the client, and the banker will obtain more benefit. Which loan would the banker recommend? Will the banker pay a cost to benefit the client by recommending Loan A? In this imaginary situation, the banker plays the advisor role, and the client plays the advice recipient role. Advisors face conflicts of interest in such situations; specifically, their interests are misaligned with those of the advice recipients (Cain et al., 2005).

Advisors are at least not always sincere when facing conflicts of interest. In other words, conflicts of interest can lead advisors to give biased or selfish advice (Moore et al., 2006). Taking the above imaginary situation as an example, the banker might recommend Loan B, which is not optimal for the client but benefits the banker more. Recent research has confirmed this. In the literature, advice-giving has typically been operationalized using monetary payoff structures, in which advisors choose between options that distribute financial outcomes between themselves and the recipient (e.g., Barneron & Yaniv, 2020; Sah, 2019). For instance, Sah et al. (2013) suggested that the majority (approximately 80%) of advisors give inferior advice. Using a different paradigm, Levine and Gomez (2021) reported that at least one-third of advisors give advice consistent with their self-interests.

Considering the prevalence of conflicts of interest and the harmfulness of selfish advice when given to advice recipients, researchers have put much effort into potential solutions. Specifically, previous research has revealed many factors that affect advisors’ tendencies to give selfish advice, such as whether conflicts of interest are disclosed (Sah, 2019), whether a third inferior alternative is introduced (Barneron & Yaniv, 2020), and whether advice recipients are identified (Sah & Loewenstein, 2012). In the literature, advice-giving has typically been operationalized using monetary payoff structures: advisors choose between options that distribute financial outcomes between themselves and the recipient (e.g., Cain et al., 2005; Moore et al., 2006; Sah, 2019). However, few studies have focused on what happens after advisors give selfish or altruistic advice. In other words, we know little about the psychological consequences of advice-giving.

A recent study of moral self-threat and how people cope with it has provided a theoretical framework to explore the psychology after advisors provide advice. This line of research suggests that people generally regard themselves as moral, while engaging in unethical behavior (Aquino & Reed, 2002). People then experience threats to their moral selves after engaging in unethical behaviors (Shalvi et al., 2015). For example, when they recall their ethical violations, people negatively update their moral self-concepts explicitly and implicitly (Barkan et al., 2012; Rothmund & Baumert, 2014). Similarly, employees experience shame after moral transgressions (Bonner et al., 2017).

In addition, people have a need to maintain positive views of their moral selves (Dunning, 2007). When experiencing threats to their moral self, people adopt various ways to cope with the threat (Barkan et al., 2015). For example, people self-servingly justify their unethical behaviors (Shalvi et al., 2015), show a greater tendency for cleansing (Zhong & Liljenquist, 2006) or compensation (Sachdeva et al., 2009), and psychologically distance themselves from their unethical behaviors (Stanley & De Brigard, 2019). More recently, researchers have suggested that people show self-serving memory errors to protect their moral selves (Wang & Shen, 2022). For instance, people actively forget details about their own moral transgressions (Kouchaki & Gino, 2016) and recall being more generous than they have been (Carlson et al., 2020).

Based on this framework of moral self-threat, we can easily predict that in advice-giving situations, advisors who give relatively more selfish advice may experience moral self-threat, considering that these selfish behaviors diverge from their responsibilities or the moral intuitions of loyalty (Graham et al., 2013). In addition, following the previous findings of self-serving memory errors (Carlson et al., 2020; Kouchaki & Gino, 2016), relatively selfish advisors may exhibit self-serving memory errors, whereas relatively prosocial advisors may not. Therefore, we first examine advisors’ evaluations of their moral self, and then examine how selfish advisors cope with the moral self-threat from the perspective of memory error.

**Figure 1:**
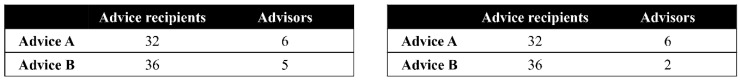
Examples of advice-giving scenarios.

We further examine the effect of prosocial cost on advisors’ moral self-evaluations and memory errors. Prosocial behaviors typically entail one paying a cost to benefit someone else (Kawamura et al., 2021). Similarly, in situations of advice-giving under conflicts of interest, altruistic advice yields benefits for advice recipients but also incurs a cost for the advisor. We define prosocial cost as this cost-benefit ratio when giving altruistic advice rather than selfish advice. Consider the two scenarios depicted in Figure 1. In the scenario on the left, compared with self-serving Advice A, altruistic Advice B results in the advisors receiving one unit less and recipient gaining four units more, yielding a prosocial cost of 0.25. Conversely, in the scenario on the right, altruistic Advice B would result in the advisors receiving four units less and the recipient gaining four units more, resulting in a prosocial cost of one.

To put it more explicitly, the higher the prosocial cost in a given scenario is, the greater the expense advisors must bear to benefit the advice recipient. This prosocial cost varies significantly across scenarios (Kawamura et al., 2021). In general, people exhibit less prosocial behavior as the cost of helping increases (Miller, 1999). However, few studies have explored the effects of prosocial cost on advisors’ moral self-evaluations and memory errors. The present research addresses this gap, potentially contributing to the fields of moral self-evaluation and self-serving memory error.

Are advisors’ evaluations of their moral self moderated by the prosocial cost of advice-giving scenarios? When the cost is low, people may feel more obligated to give altruistic advice (Tomasello, 2020). Accordingly, one may predict that selfish advisors would experience greater moral self-threat when the prosocial cost is low. However, the present research mainly focuses on the relationship between the number of prosocial advice choices and the advisors’ moral self-evaluations. Considering that people see themselves as more moral when they engage in more prosocial behavior (Zlatev et al., 2020), we predict that the more prosocial advice the advisors give, the higher moral self-evaluations they will report regardless of whether the prosocial cost of advice-giving scenarios is high or low. Consistently, the advisors giving less prosocial advice will always engage in self-serving memory errors to cope with their moral self-threat. In the present research, we ask the participants to play the advisor role, give advice, and then recall the advice they give. We specifically predict that relatively selfish advisors recall giving more altruistic advice than they actually gave.

However, the memory errors of relatively altruistic advisors may be moderated by the prosocial cost of advice-giving scenarios. We propose that this moderation can be understood through a regret regulation mechanism (Zeelenberg & Pieters, 2007). Under high prosocial cost, giving altruistic advice requires a substantial personal sacrifice, which can evoke post-decisional regret. This negative emotional state is one that individuals are motivated to attenuate (Coricelli et al., 2005). By misremembering their actions as less altruistic than they actually were, these advisors indirectly minimize the perceived magnitude of their personal sacrifice, thereby reducing the regret associated with their costly prosocial advice. Under low prosocial cost, in contrast, the personal sacrifice required for altruistic advice is minimal. This elicits little regret and, consequently, no motivation for biased recall. Thus, we predict that relatively altruistic advisors will show self-defeating memory errors under high prosocial cost; specifically, they recall less prosocial advice than was actually given, but they will show no memory bias under low prosocial cost.

In short, the present research examines the psychological consequences of advice-giving, with a specific focus on how these advisors evaluate their moral selves and potential memory errors. More importantly, we delve into the impact of prosocial cost on advisors’ moral self-evaluations and memory errors. Based on the aforementioned theoretical framework, we propose the following hypotheses:

**Hypothesis** **1.** *The number of prosocial advice choices is positively associated with advisors’ moral self-evaluations, regardless of whether the prosocial cost is high or low.*

**Hypothesis** **2.** *Relatively selfish advisors are expected to exhibit self-serving memory errors, specifically recalling giving more prosocial advice than they actually gave, irrespective of the prosocial cost.*

**Hypothesis** **3.** *The memory error of relatively altruistic advisors is expected to depend on prosocial cost. Under high prosocial cost, they are predicted to show self-defeating memory errors (i.e., recalling less prosocial advice than was actually given); under low prosocial cost, they are predicted to show no memory error.*

We conducted three studies to test these hypotheses. In Study 1, we introduced advice-giving scenarios with both high and low prosocial costs. However, to avoid the potential confounding effects of including two different prosocial cost scenarios within a single study, for Study 2 and Study 3 we exclusively employed scenarios with either low or high prosocial costs, respectively, to validate the strength of the findings. Furthermore, drawing from previous research (Saucet & Villeval, 2019), we categorized high prosocial cost as 1 and low prosocial cost as 0.25.

## 2. Study 1: Scenarios of Advice-Giving with Both High and Low Prosocial Costs

### 2.1. Method

#### 2.1.1. Participants

All studies were conducted in China. For sampling convenience, participants were recruited from the authors’ normal university. Specifically, we posted recruitment advertisements through the university’s participant recruitment system, and students voluntarily signed up to participate. The system exclusively serves undergraduate and postgraduate students of this university. The recruitment advertisement described the study in neutral terms as a study on decision-making, without mentioning advice-giving, moral evaluation, or the selfish versus altruistic nature of the advice. This was to avoid priming participants toward any particular self-presentation goal prior to the experimental session.

We finally recruited one hundred and eight participants. Five of them exited due to computer issues, yielding a final sample size of 103 (*M*_age_ = 20.80, *SD*_age_ = 1.78, and 85 females). We offered a compensation of ¥5 for the participants. In addition, participants could receive additional bonuses based on their decisions in the study.

#### 2.1.2. Design

The predictor variable in this study was the number of prosocial advice choices participants made, treated as a continuous variable. The first outcome variable was the participants’ evaluation of their moral self, which was measured using the moral self-concept scale (Kouchaki & Gino, 2016).

The second outcome variable was memory errors. Following Carlson et al. (2020), memory errors were determined by calculating the discrepancy between the number of instances of altruistic advice the participants recalled they gave and the actual number of instances of altruistic advice they did give. Memory error scores not significantly deviating from zero indicate that the participants did not exhibit significant memory bias. Scores significantly greater than zero indicate that participants overestimated the frequency of their altruistic advice, which is indicative of a self-serving memory error. Conversely, scores less than zero indicate that participants significantly underestimated their altruistic advice frequency, which is indicative of a self-defeating memory error.

#### 2.1.3. Procedure

The participants arrived at the laboratory in pairs and completed the study following the instructions.

*Assigning the role.* First, we informed the participants that they would complete a game in which they played the role of advisor or advice recipient and that their roles were determined based on a computer program. The program assigned all the participants as advisors.

*Giving advice.* Next, we asked the participants to give their advice. Specifically, we told the participants that as advisors, they would choose between two pieces of advice and give one to the advice recipient. Taking the scenario on the left in Figure 1 as an example, as a result of Advice A, they would receive six tokens and the advice recipient would receive 32 tokens. However, as a result of Advice B, they would receive five tokens and the advice recipient would receive 36 tokens.

Notably, in these advice-giving scenarios, one of the pieces of advice was relatively selfish, allowing the advisors to get more but the advice recipients to get less. The other advice was relatively altruistic, benefiting the advice recipients but costing the advisors. Taking the scenario on the left in Figure 1 as an example, Advice B was altruistic, whereas Advice A was selfish.

There were six trials of advice-giving, which were presented randomly. That is, the participants gave advice six times. In half of the trials, Advice A was altruistic. However, in the other half, Advice A was selfish. The participants gave Advice A by pressing the “F” key and gave Advice B by pressing the “J” key.

Following Saucet and Villeval (2019), there were two prosocial costs in Experiment 1 (i.e., 0.25 and 1). There were three trials of advice-giving scenarios for each prosocial cost (for details, see the Supplemental Materials).

Notably, the advice given was related to the participants’ additional bonus. That is, each token they received could be exchanged for ¥0.01 after the study. To prevent the possible effect of social approval, we informed the participants that the advice recipients could see only their own benefits. In other words, the advice recipients did not know the advisors’ benefits or the advice the advisor did not give. Furthermore, we paid the participants electronically after they left.

*Moral self-evaluation.* After giving advice, moral self-evaluation was assessed using the ten-item moral self-concept scale (Kouchaki & Gino, 2016). Participants were asked to indicate how they felt about themselves at that moment on a series of traits (e.g., moral, generous, and loyal) using a seven-point Likert scale (1 = not at all; 7 = very much).

To our knowledge, this scale has not been validated for use in China. Therefore, we conducted a post hoc exploratory factor analysis. The Kaiser–Meyer–Olkin (KMO) measure was 0.89, and Bartlett’s test of sphericity was significant, with χ^2^(45) = 826.85 and *p* < 0.001, indicating that the data were suitable for factor analysis. Using principal component analysis, a single factor with an eigenvalue greater than one was extracted, accounting for 62.93% of the total variance. All items loaded strongly on this primary factor, with loadings exceeding 0.60. Subsequently, the reliability of this factor was assessed, yielding a Cronbach’s alpha of 0.93, indicating good internal consistency. These results suggested this scale was valid.

*Filler task.* The participants then completed a filler task designed to displace the advice-giving decisions from their short-term memory, thereby creating the conditions for memory error to occur (Tasimi & Johnson, 2015). Given that information in short-term memory decays rapidly when active rehearsal is prevented (Atkinson & Shiffrin, 1971), we employed a maze-solving task lasting approximately eight minutes—a duration far exceeding the temporal window of short-term retention. This ensured that the subsequent recall task captured long-term memory reconstruction rather than residual working memory traces. As in Saucet and Villeval (2019), the participants solved the mazes on paper.

*Advice recall.* We presented the six advice-giving scenarios at random again and asked the participants to recall the advice they had previously given. Notably, before this, we had never told the participants that they would recall their advice.

### 2.2. Results

Prior to testing our primary hypotheses, we examined whether gender influenced the two core dependent measures (i.e., moral self-evaluation and memory error) across all three studies. No significant effects of gender were found in any study (all *p* > 0.15). We therefore did not include gender as a covariate in the analyses reported below. Notably, controlling for gender did not meaningfully alter the significance or pattern of any of the reported results.

#### 2.2.1. Moral Self-Evaluation

To test Hypothesis 1, we analyzed the relationship between prosocial advice-giving and average moral self-evaluation (α = 0.93) using Pearson correlation and simple linear regression. The results showed that the number of prosocial advice choices was positively correlated with and significantly predicted moral self-evaluation (*r* = 0.20, *p* = 0.045; *b* = 0.14, *SE* = 0.08, and *p* = 0.045), supporting Hypothesis 1.

#### 2.2.2. Memory Errors in Scenarios of Advice-Giving with Either High or Low Prosocial Costs

To test Hypotheses 2 and 3, we employed a floodlight analysis (Spiller et al., 2013). In contrast to traditional spotlight tests that evaluate effects at arbitrarily selected predictor values (e.g., mean ± 1 *SD*), floodlight analysis identifies the region(s) of significance in which the predicted outcome is statistically different from zero across the entire observed range of the predictor.

Specifically, we regressed memory error on the continuous number of prosocial advice choices. Using SPSS 26.0’s Save option in the Linear Regression dialog, we saved the following cases: (a) the unstandardized predicted values (PRE_1), and (b) the 95% confidence intervals for the mean prediction (LMCI_1 and UMCI_1). Consequently, we obtained a predicted memory error and its 95% confidence interval at each integer level of prosocial advice choices. A predicted memory error was considered significantly different from zero at a given prosocial value if the corresponding 95% confidence interval did not contain zero. By inspecting the confidence intervals at each integer level, we identified the range of prosocial advice choices for which the memory error was significantly positive (self-serving bias) and the range for which it was significantly negative (self-defeating bias). Notably, all the predicted memory error along with its 95% confidence intervals were tabulated in the Supplemental Materials.

The results showed that, when the prosocial cost was 0.25, the number of prosocial advice choices significantly and negatively predicted the memory errors: *b* = −0.29, *SE* = 0.09, *p* = 0.001, and *R*^2^ = 0.10. Additionally, memory error was significantly positive (self-serving) when the number of prosocial advice choices was zero or one (*N* = 47); but was not significantly different from zero when the number of prosocial advice choices was two or three (*N* = 56).

When the prosocial cost was one, the number of prosocial advice choices was also significantly and negatively predicted the memory errors: *b* = −0.37, *SE* = 0.07, *p* < 0.001, and *R*^2^ = 0.20. In addition, memory error was significantly positive (self-serving) when the number of prosocial advice choices was 0 (*N* = 35), but was significantly negative (self-defeating) when the number of prosocial advice choices was one, two, or three (*N* = 68). These findings confirmed the Hypothesis 2 and 3.

#### 2.2.3. Sensitivity Power Analysis

We conducted post hoc sensitivity power analyses using G*Power 3.1.9 (Faul et al., 2007) to determine the minimum effect sizes detectable with our sample given 80% statistical power (α = 0.05).

For Hypothesis 1 (tested via Pearson correlation), with *N* = 103, the minimum detectable correlation was ∣*r*∣ = 0.27. The observed correlation between the number of prosocial advice choices and moral self-evaluation was *r* = 0.20, slightly below this threshold, suggesting limited power for this specific effect.

For Hypotheses 2 and 3 (tested via simple linear regression), the minimum detectable effect size with one predictor was *f*^2^ = 0.08. In the low prosocial cost condition (cost = 0.25), the model was significant, *R*^2^ = 0.10, yielding *f*^2^ = 0.11, a value well above the threshold. In the high prosocial cost condition (cost = 1), *R*^2^ = 0.20, yielding *f*^2^ = 0.25, which also exceeded the threshold. These results indicate that our sample size was adequate for detecting the effects central to Hypotheses 2 and 3.

### 2.3. Discussion

Study 1 provided initial evidence for all three hypotheses using a within-subjects design in which participants encountered both low and high prosocial cost scenarios. The results confirmed that participants who gave fewer prosocial advice choices reported lower moral self-evaluations, supporting Hypothesis 1. Regarding memory errors, participants who gave relatively few prosocial advice choices exhibited self-serving memory biases irrespective of whether prosocial cost was high or low, consistent with Hypothesis 2. In contrast, participants who gave relatively more prosocial advice choices showed a pattern moderated by prosocial cost: under a low cost, they displayed no significant memory bias, whereas under a high cost, they exhibited self-defeating memory errors, recalling their behavior as less altruistic than it actually was. This pattern is consistent with Hypothesis 3.

However, Study 1 had two methodological limitations that warranted further investigation. First, each prosocial cost condition included only three advice-giving trials, which may have introduced random error due to the insufficient sampling of participants’ behavior. Second, the within-subjects manipulation of prosocial cost, while efficient, may have produced carryover effects: participants’ moral self-evaluations after giving selfish advice under low cost could influence their subsequent responses under high cost, or vice versa.

To address these concerns, Studies 2 and 3 employed a between-subjects manipulation of prosocial cost and increased the number of trials to twelve per condition (Saucet & Villeval, 2019). Study 2 focused exclusively on low prosocial cost scenarios, while Study 3 focused exclusively on high prosocial cost scenarios. This design isolates the effects of each cost condition without the potential confound of within-subject carryover, thereby providing a cleaner test of all three hypotheses.

## 3. Study 2: Scenarios of Advice-Giving with Low Prosocial Costs

### 3.1. Method

#### 3.1.1. Participants

We determined the sample size as in Study 1 and finally recruited one hundred seven participants from a university as in Study 1. Two of the individuals were ultimately not included due to computer issues, yielding a final sample size of 105 (*M*_age_ = 20.15, *SD*_age_ = 1.38, and 84 females). We paid the participants as in Study 1.

#### 3.1.2. Design and Procedure

The design and procedure mirrored those of Study 1, with the exception that the participants were presented with 12 advice-giving scenarios (for details, see the Supplemental Materials). Furthermore, the prosocial cost associated with these scenarios was set at 0.25.

### 3.2. Results

#### 3.2.1. Moral Self-Evaluation

To test Hypothesis 1, we analyzed the average moral self-evaluation (α = 0.96). The results showed that the number of prosocial advice choices was positively correlated with and significantly predicted moral self-evaluation (*r* = 0.21, *p* = 0.03; *b* = 0.07, *SE* = 0.03, and *p* = 0.03), confirmed Hypothesis 1.

#### 3.2.2. Memory Error

We analyzed memory error as in Study 1 to test Hypothesis 2 and 3. The results showed that the number of prosocial advices significantly and negatively predicted the memory errors, *b* = −0.11, *SE* = 0.04, *p* = 0.009, and *R*^2^ = 0.07. Additionally, memory error was significantly positive (self-serving) when the number of prosocial advice choices was between zero and five (*N* = 38), but indicated no significant deviation from zero when the number of prosocial advice choices was six or more (*N* = 67).

#### 3.2.3. Sensitivity Power Analysis

With *N* = 105, the minimum detectable correlation (80% power; α = 0.05) was ∣*r*∣ = 0.27. For Hypothesis 1, the observed correlation between prosocial advice choices and moral self-evaluation was *r* = 0.21, again slightly below the threshold. For Hypotheses 2 and 3, the minimum detectable *f*^2^ for a one-predictor regression was 0.076. The regression of memory errors on prosocial advice choices yielded *R*^2^ = 0.07, *f*^2^ = 0.075, which closely approximated the threshold.

### 3.3. Discussion

Study 2 replicated the primary findings of Study 1 in advice-giving scenarios with low prosocial cost. Specifically, advisors who gave relatively few prosocial advice choices reported lower moral self-evaluations than did those who gave more prosocial advice choices, a pattern consistent with Hypothesis 1 and moral self-threat. Regarding memory errors, the floodlight analysis confirmed that participants who gave relatively few prosocial advice choices (between zero and five out of twelve trials) exhibited significant self-serving memory biases, consistent with Hypothesis 2. In contrast, participants who gave relatively many prosocial advice choices (six or more) showed no significant memory bias, consistent with Hypothesis 3 under low prosocial cost. In Study 3, our objective was to reproduce the principal findings of Study 1 but in the context of advice-giving situations with high prosocial cost.

## 4. Study 3: Scenarios of Advice-Giving with High Prosocial Costs

### 4.1. Method

#### 4.1.1. Participants

We recruited 111 participants from a university (*M*_age_ = 20.15, *SD*_age_ = 1.38, and 93 females). We paid them as in Study 1.

#### 4.1.2. Design and Procedure

The design and procedure of Study 3 were similar to those of Study 1 except that Study 3 included 12 advice-giving scenarios (for details, see the Supplemental Materials), and the prosocial cost of these scenarios was high (i.e., one).

### 4.2. Results

#### 4.2.1. Moral Self-Evaluation

We analyzed the average moral self-evaluation (α = 0.96) to examine Hypothesis 1. The results revealed that the number of prosocial advice choices was positively correlated with and significantly predicted moral self-evaluation (*r* = 0.39, *p* < 0.001, and *b* = 0.15, *SE* = 0.03, and *p* < 0.001), supporting Hypothesis 1.

#### 4.2.2. Memory Error

The results suggested that the number of prosocial advices significantly and negatively predicted the memory errors, *b* = −0.17, *SE* = 0.05, *p* = 0.001, and *R*^2^ = 0.09. Additionally, memory error was significantly positive (self-serving) when the number of prosocial advice choices was between zero and two (*N* = 36), indicated no significant deviation from zero when the number of prosocial advice choices was between three and seven (*N* = 59), and was significantly negative (self-defeating) when the number of prosocial advice choices was eight or more (*N* = 16).

#### 4.2.3. Sensitivity Power Analysis

With *N* = 111, the minimum detectable correlation (80% power; α = 0.05) was ∣*r*∣ = 0.26. For Hypothesis 1, the observed correlation between prosocial advice choices and moral self-evaluation was *r* = 0.39, comfortably exceeding the threshold. A supplementary internal meta-analysis combining the three correlations using Fisher’s method yielded a pooled *r* = 0.27, 95% CI [0.17, 0.37], and *p* < 0.001, confirming that the positive association between prosocial advice choices and moral self-evaluation was reliable across the full dataset.

For Hypotheses 2 and 3, the minimum detectable *f*^2^ for a one-predictor regression was 0.07. The regression of memory errors on prosocial advice choices yielded *R*^2^ = 0.09 and *f*^2^ = 0.10, exceeding the threshold. These results indicate that Study 3 was adequately powered to detect the effects of primary interest.

#### 4.2.4. Analysis of the Type of Advisor × Prosocial-Cost Interaction Across All Three Studies

To provide a principled test of the interaction between advisor type and prosocial cost, we conducted a logistic mixed-effects model on the trial-level data. The analysis was performed in R using the lme4 package (Brooks et al., 2017), fitting a generalized linear mixed-effects model with a logistic link function. The advisor type (selfish vs. altruistic, determined by the advice actually given on a given trial) and prosocial cost (0.25 vs. 1) were entered as fixed effects, with the presence versus absence of memory error as the binary dependent variable. Random intercepts for participants were included to account for the non-independence of trials within individuals. To maximize statistical power and provide the most comprehensive and generalizable test of the interaction, we integrated the data from all three studies, resulting in a total of 319 participants who completed 3210 trials.

Notably, the raw regression coefficients (*b*) from a logistic mixed-effects model are on the log-odds scale, which is not readily interpretable as a standardized effect size. We therefore computed the effect size *r* for each fixed effect from the Wald *z*-statistic. These *r* values can be interpreted similarly to a Pearson correlation, with larger absolute values indicating stronger effects. For completeness, we also report the raw coefficients *b*, their standard errors, and the *z*-statistics. All confidence intervals reported below are the 95% confidence intervals of *r*.

The results revealed a significant main effect of advisor type; that is, altruistic advisors were overall more likely to exhibit memory errors: *b* = 0.21, *SE* = 0.11, *z* = 1.96, *p* = 0.050, *r* = 0.12, and 95% CI [0.00, 0.23]. The main effect of prosocial cost was not significant: *b* = −0.18, *SE* = 0.14, *z* = −1.28, *p* = 0.20, and *r* = −0.10, 95% CI [−0.24, 0.05]. Critically, the two-way interaction was significant: *b* = 1.49, *SE* = 0.22, *z* = 6.91, *p* < 0.001, *r* = 0.63, and 95% CI [0.51, 0.73].

Simple effects analyses further showed that for selfish advisors, the effect of prosocial cost was significant: a higher cost was associated with a lower probability of exhibiting memory errors: *b* = −0.92, *SE* = 0.17, *z* = −5.41, *p* < 0.001, *r* = −0.45, and 95% CI [−0.57, −0.31]. For altruistic advisors, the effect of prosocial cost was also significant, but in the opposite direction; higher cost was associated with a higher probability of exhibiting memory errors: *b* = 0.57, *SE* = 0.18, *z* = 3.18, *p* = 0.001, *r* = 0.30, and 95% CI [0.12, 0.45].

### 4.3. Discussion

Study 3, which focused on advice-giving scenarios with high prosocial costs, successfully replicated the primary findings of Study 1. Specifically, advisors who gave fewer prosocial advice choices reported lower moral self-evaluations than did those who gave more prosocial advice choices, a pattern consistent with moral self-threat. Furthermore, participants who gave relatively few prosocial advice choices exhibited significant self-serving memory biases. In contrast, the floodlight analysis provided statistically significant evidence consistent with Hypothesis 3: participants who gave the most prosocial advice (eight choices or more) showed a significant self-defeating memory bias. However, we note that only 14.4% of the sample fell within this range, and future research with larger samples is needed to establish the robustness of this effect.

The logistic mixed-effects analysis integrating trial-level data from all three studies provided a formal, principled test of the advisor type × prosocial cost interaction. The significant interaction confirmed that the type of memory error depends jointly on whether the advice given was selfish or altruistic and on the prosocial cost of the scenario. Specifically, in trials where altruistic advice was given, memory errors were more likely under high prosocial cost than under low prosocial cost, consistent with Hypothesis 3. In trials where selfish advice was given, the reverse pattern emerged, a finding we return to in the General Discussion.

## 5. General Discussion

The present research focused on the psychological consequences of advice-giving. Across three studies, we provided consistent evidence for all three hypotheses. We consistently found that advisors who gave fewer prosocial advice choices reported lower moral self-evaluations than did those who gave more prosocial advice choices in both the high- and low-prosocial cost scenarios, a pattern consistent with moral self-threat. In addition, we found that advisors who gave relatively few prosocial advice choices showed self-serving memory errors in both high- and low-prosocial cost scenarios. That is, they remembered giving more prosocial advice than they actually did. However, among advisors who gave relatively many prosocial advice choices, memory errors were moderated by prosocial cost. In the low prosocial cost scenarios, there were no memory errors. However, in the high prosocial cost scenarios, the opposite effect was observed for self-serving memory errors. Specifically, they remembered giving less prosocial advice than they actually did.

Advice-giving under conflicts of interest has become an increasingly popular topic in both psychological and ethical studies. Previous research has revealed several factors that may affect advisors’ decisions (Barneron & Yaniv, 2020; Sah, 2019). However, few studies have focused on the psychology after advisors provide advice. Based on the theory of moral self-threat and how people cope with it, the present research examined advisors’ evaluations of their moral self and memory errors. These findings contribute to the field of advice-giving by suggesting that giving selfish advice is associated with lower moral self-evaluations, a pattern consistent with moral self-threat. In addition, we found that selfish advisors recalled giving more prosocial advice than they actually did, which may lessen the moral self-threat they experience.

It is worth noting that participants in our studies gave altruistic advice at rates that appear higher than some prior estimates. Sah et al. (2013) suggested that approximately 80% of advisors give selfish advice under conflicts of interest. In our studies, however, across all 319 participants and 3210 trials, 1404 trials involved altruistic advice (43.8%), meaning that selfish advice was given on the remaining 56.2% of trials—substantially lower than the 80% reported by Sah et al. (2013). Importantly, these data were obtained under neutral recruitment conditions: participants were told they were taking part in “a study on decision-making”, with no mention of advice-giving, moral evaluation, or the selfish versus altruistic nature of the choices. This neutral framing rules out the possibility that our findings reflect self-selection by individuals who were particularly motivated to be “good advisors.”

The discrepancy between our overall rate of altruistic advice and the 80% selfish advice estimate from Sah et al. (2013) likely reflects differences in the effective prosocial cost embedded in the respective paradigms. When the personal sacrifice required to act altruistically is small, as in our low-cost conditions, participants are far more willing to give altruistic advice. This pattern is also observed by Barneron and Yaniv (2020), who reported that conflicted advisors gave approximately 70% prosocial recommendations in comparable conditions. This cost-dependent variability underscores the value of systematically manipulating prosocial cost, as we have done, and cautions against treating the prevalence of selfish advice-giving as a fixed empirical constant.

These findings offer novel avenues for interventions aimed at encouraging advisors to provide more prosocial advice. For instance, research has shown that to cope with the possible moral self-threat, people may avoid lying too much (Mazar et al., 2008) or be more likely to return a lost purse (Cohn et al., 2019). Our work reveals that advisors who give relatively selfish advice similarly report lower moral self-evaluations, a pattern consistent with moral self-threat. Future practitioners might therefore leverage this insight by guiding these advisors to focus more consciously on their moral standards (Mazar et al., 2008), thereby nudging them toward more altruistic recommendations. Furthermore, given that self-serving memory bias may help mitigate the moral self-threat experienced by selfish advisors, an alternative approach worth exploring would be to disrupt such biased memory reconstruction. By reducing advisors’ reliance on self-justifying memory distortions, we might effectively diminish their propensity to repeatedly provide self-serving advice.

The present research also contributes to the field of moral self-threat and how people cope with it at least in the following aspects. First, the advice-giving context under conflicts of interest studied in our manuscript represents a specific instance of the broader dilemma involving trade-offs between self-interest and the interests of others, a theme extensively explored in simple economic games. Researchers in social psychology and behavioral ethics have taken the moral self into consideration. However, the specific moral values involved may vary across different situations. Previous research has focused mainly on the taking game which mainly involve the moral values of honesty (Mazar et al., 2008), and allocation game in which fairness value involve (Carlson et al., 2020; Saucet & Villeval, 2019). We extended this line of research to advice-giving under conflict of interest, which involve moral principle of loyalty or responsibility, and suggest that giving selfish advice is associated with lower moral self-evaluations, and with self-serving memory errors. From this perspective, our study broadens the scope of research on moral self-threat by demonstrating that the theoretical framework remains applicable in contexts involving the principle of loyalty.

Second, we explored the effect of prosocial cost on self-serving memory error and revealed that among selfish trials memory errors were more likely under a low cost than under a high cost. This pattern is consistent with a self-justification account (Shalvi et al., 2015). Specifically, when prosocial cost is high, giving selfish advice is relatively easy to rationalize as the personal sacrifice required to act altruistically is substantial, thereby posing a modest threat to the moral self and reducing the need for biased recall. When the prosocial cost is low, however, prioritizing a small personal gain over a large benefit for the recipient is difficult to justify, heightening moral self-threat and motivating self-serving memory distortions to restore a positive self-image (Mazar et al., 2008).

Third and more importantly, we found that advisors who gave a lot of altruistic advice remembered giving less than they actually did in the high but not low prosocial cost scenarios. Trial-level analysis also showed that, in trials where altruistic advice was given, these self-defeating memory errors were more likely to occur under a high prosocial cost than under a low prosocial cost. To our knowledge, we are the first to report this self-defeating memory error. As we mentioned previously, regret regulation may account for the prosocial advisor’s self-defeating memory errors. That is, in high prosocial cost scenarios, giving prosocial advice may result in greater regret (Coricelli et al., 2005; Zeelenberg & Pieters, 2007). The altruistic advisors then show self-defeating memory errors to mitigate this negative feeling.

Besides this regret regulation account, altruistic advisors’ self-defeating memory errors may also result from their rational inference processes. That is, people infer their past behaviors based on their beliefs about what they normally do (Carlson et al., 2020). Previous research has suggested that this rational inference process can explain some social comparative biases, such as above-average effects (Chambers & Windschitl, 2004; Kruger, 1999). Similar logic may also apply to our research. Specifically, people may hold the belief that they will perform ordinary but not extreme prosocial behaviors. Accordingly, altruistic advisors will show no memory errors in low prosocial cost scenarios but will show self-defeating memory errors in the high prosocial cost scenarios.

It is also worth considering whether this self-defeating memory error observed among altruistic advisors under high prosocial cost reflects socially desirable responding rather than genuine memory distortion. Because particularly generous behavior can be viewed as norm-deviant when the cost of helping is high (Berman & Silver, 2022; Kawamura & Kusumi, 2020), participants may have strategically under-reported their altruistic choices to align with perceived social norms. Several features of our design reduce this possibility: the advice-giving context was fully anonymous, recipients never saw the advisor’s payoffs or foregone options, and the recall task was presented as a surprise memory test. Nevertheless, the present data cannot definitively rule out a social desirability account. Future research should employ incentivized memory paradigms (Carlson et al., 2020), in which participants are rewarded for accurate recall, to disentangle motivated misreporting from genuine memory errors.

Several limitations and future directions merit mention. First, regarding internal validity, our design did not experimentally manipulate advisors’ behavior, so the observed associations remain correlational and cannot support causal inference. For instance, individuals with chronically lower moral self-regard may simply be more inclined to give selfish advice. Relatedly, the moral self-concept scale (Kouchaki & Gino, 2016) was used without prior validation in a Chinese-speaking population. Although internal reliability was high across studies (α = 0.93–0.96), the psychometric evidence was derived from the same samples used for hypothesis testing and therefore cannot be considered independent. Future research should address these concerns by experimentally manipulating advisor behavior, measuring relevant individual differences (e.g., social value orientation; Murphy et al., 2011) as covariates, and validating the scale in an independent Chinese-speaking sample with systematic forward-back translation and confirmatory factor analysis.

Second, the generalizability of our findings is constrained by both the laboratory paradigm and the sample. Our token allocation task, while offering experimental control, omits features of real-world advisory contexts such as ongoing professional relationships, reputational stakes, and legal obligations like fiduciary duty. Future work should test whether these dynamics moderate the psychological consequences we observed, for example, through role-play scenarios with professional advisors (e.g., financial consultants and medical practitioners). Additionally, all participants were university students from a single institution in China, with a marked gender imbalance (approximately 80% female). Given that our study was conducted in a relatively collectivist cultural context, where norm violations tend to evoke stronger condemnation (Stamkou et al., 2019) and extreme altruism may be viewed less favorably (Kawamura & Kusumi, 2020), cross-cultural replication in more individualistic settings is warranted.

Third, although post hoc sensitivity power analyses confirmed that our samples were generally adequate for detecting the primary hypothesized effects, the observed effect sizes were small to medium. The self-defeating memory error was supported by the floodlight analysis but emerged in a relatively small subset of participants (e.g., 16 of 111 in Study 3). Larger samples are needed to more precisely characterize the prevalence and robustness of this novel phenomenon.

Finally, although our theoretical framework emphasizes regret regulation as the primary mechanism underlying self-defeating memory errors, we did not directly measure post-decisional regret in any of the three studies. Our account therefore remains post hoc, and the observed memory biases may also be consistent with other mechanisms, such as the rational inference processes we discuss above. Future research should incorporate validated measures of regret (e.g., Marcatto & Ferrante, 2008) to provide direct evidence for the proposed affective pathway.

In conclusion, this study was conducted to examine the psychological consequences of advice-giving and yielded three primary findings. First, advisors who gave fewer prosocial advice choices reported lower moral self-evaluations than those who gave more prosocial advice choices, a pattern consistent with moral self-threat. Second, these selfish advisors showed self-serving memory biases, inaccurately recalling that they had given more altruistic advice than they actually had. Finally, advisors who gave relatively many prosocial advice choices showed no memory errors when prosocial cost was low, but displayed self-defeating memory biases when prosocial cost was high, inaccurately recalling that they had given less altruistic advice than they actually had. These findings enhance our understanding of the psychological processes that occur after advisors give advice and highlight the novel phenomenon of self-defeating memory error.

## Data Availability

All data in this study are available at https://osf.io/eh3xn/, accessed on 30 April 2026.

## Supplementary Materials

All Supplemental Materials in this study are available at https://osf.io/eh3xn/.
